# 人工智能在血液肿瘤精准诊疗中的应用进展

**DOI:** 10.3760/cma.j.cn121090-20241022-00409

**Published:** 2025-02

**Authors:** 皓旭 杨, 杰 熊, 维莅 赵

**Affiliations:** 上海交通大学医学院附属瑞金医院血液科，医学基因组学国家重点实验室，上海血液学研究所，上海 200025 Department of Haematology, State Key Laboratory of Medical Genomics, Shanghai Institute of Haematology, Ruijin Hospital Affiliated to Shanghai Jiao Tong University School of Medicine, Shanghai 200025, China

## Abstract

血液肿瘤属于高度异质性的恶性疾病，其生物学特征复杂且临床表现多样化，实施精准诊疗显得尤为重要。为进一步提升诊疗精度、提高预后预测准确性、推进个性化医疗，人工智能在血液肿瘤中的应用日趋广泛。本文总结了近五年来人工智能在血液肿瘤的诊断、治疗以及预后预测等方面的应用，对其优势和不足进行了探讨。人工智能可有效提高血液肿瘤精准诊疗的能力，但面临数据质量欠佳、模型可解释性较差以及临床转化有限等瓶颈问题。未来可通过建立临床数据标准、融合多模态信息进行精准诊疗和预后预测、开展模型算法系统化的临床验证等，从而加快人工智能在血液肿瘤临床中的推广和应用。

## Abstract (en)

Hematological malignancy is a highly heterogeneous disease with complex biological characteristics and diverse clinical manifestations. Therefore, precise diagnosis and treatment are crucial and urgently needed. To further improve the accuracy of diagnosis and prognostication and to promote personalized therapy, artificial intelligence (AI) has been increasingly used. This study reviewed literature published in the last 5 years and summarized the application, benefits, and drawbacks of AI in the diagnosis, treatment, and prognosis of hematologic malignancies. Although AI can effectively improve the accuracy of diagnosis and therapy, low-quality data, poor interpretability of the model, and limited clinical transformation have impeded its popularization and application. In the future, the clinical application of AI in hematologic malignancy can be accelerated by establishing standards for clinical data processing, integrating multimodal information for accurate diagnosis and prognostication, and conducting systematic clinical verification of model algorithms.

血液肿瘤是一类起源于造血器官并主要累及造血系统的高度异质性恶性疾病[1]–[2]，主要类型包括由造血干细胞恶性克隆性增殖引发的白血病[3]–[5]、起源于淋巴结及淋巴组织的淋巴瘤[6]–[7]，以及单克隆浆细胞在骨髓中恶性增生并广泛浸润引发的多发性骨髓瘤（MM）[8]–[9]。上述疾病具有复杂的生物学特征和多样的临床表现，对其进行精准诊断与治疗始终是医学领域面临的重大挑战。近年来，随着诊疗技术的进步，来自于不同仪器和临床记录的诊疗数据，呈现出数据量大、类型多样等特点。传统依靠医师经验的诊断模式，存在主观差异性大，诊断效率低，判断的系统性、科学性和准确性难以保证等问题。鉴于此，如何利用人工智能（AI）技术提升诊断效率和精度，优化治疗决策，改善临床疗效成为目前研究的热点问题。

AI通过模拟人类智能来提供自动化解决方案，提高工作效率并拓展人类的认知能力[10]–[12]。机器学习是人工智能的一个重要部分，是一种利用计算机从数据中产生“模型”的方法[13]。机器学习一般包括监督学习（如回归分析）、无监督学习（如聚类）、强化学习等。深度学习作为机器学习的一个分支，近年来，深度学习发展迅猛、应用广泛。深度学习是指以复杂神经网络为模型的机器学习[14]，代表性的技术有卷积神经网络（CNN）、循环神经网络（RNN）等，目前广泛应用于图像识别[15]、语音识别[16]和自然语言处理[17]等方面。大语言模型（LLM）是使用大量文本数据训练的深度学习模型[18]，它能够高效理解和生成自然语言。随着数据的增长和算力的提升，LLM发展迅速，并逐步融合图像、声音等数据向多模态大模型方向发展。多模态学习是一种能够同时处理文本、图像、语音等多种数据类型的AI技术[19]。通过对不同类型数据的融合与分析，提高了AI的综合推理和决策能力，是人工智能的一个重要方向。

目前，AI正广泛应用于各个学科[10],[20]，在医学领域，AI主要应用于医学图像分析[21]–[22]、疾病预测和诊断[23]–[25]、自然语言处理（NLP）[26]–[28]和临床决策支持系统（CDSS）[29]–[31]等方面。在血液肿瘤方面，AI技术已逐步应用于精准诊疗和预后预测中，表1展示了部分代表性工作。本文以数据为依据、AI技术为核心总结近5年来AI在血液肿瘤的精准诊断、治疗和预后预测方面的应用，梳理当前应用中所面临的困难挑战与不足，探讨未来的发展方向，为AI应用于血液肿瘤提供重要的参考依据。

**表1:** 人工智能在血液肿瘤应用中的部分代表性工作

应用类型	参考文献	应用	数据	模型
精准诊断	Eckardt等[32]	AML患者和健康骨髓捐献者	图像数据（骨髓涂片图像数据）	卷积神经网络
精准诊断	李兰兰等[33]	诊断DLBCL患者的骨髓受累情况	多模态数据（PET和CT图像数据）	卷积神经网络
精准诊断	Xiong等[34]	区分NKTCL的分子亚型	分子数据（RNA-seq数据）	聚类算法
精准治疗	Jemaa等[35]	自动对PET/CT数据进行代谢反应评估	图像数据（PET/CT图像数据）	深度学习算法
精准治疗	Shain等[36]	为多发性骨髓瘤患者临床决策提供支持	多模态数据（临床数据和分子分析数据）	强化学习算法
精准治疗	Lee等[37]	自动抽取临床试验摘要中安全性和有效性信息	文本数据（临床试验摘要）	大语言模型
预后预测	Jiang等[38]	预测DLBCL患者的生存情况	图像数据（PET图像数据）	卷积神经网络
预后预测	He等[39]	构建DLBCL患者的预后风险模型	分子数据（mRNA数据）	回归分析

**注** AML：急性髓系白血病；DLBCL：弥漫大B细胞淋巴瘤；NKTCL：自然杀伤T细胞淋巴瘤

一、AI在血液肿瘤精准诊断方面的应用

AI在血液肿瘤诊断中的应用主要集中在基于图像数据和分子数据的分析诊断。在图像分析方面，AI促进了图像数据分析从单一模态向多模态融合，同时模型的泛化能力和临床适用性也得到了提高；在分子数据分析方面，AI深入解析了血液肿瘤异质性特征，优化了分子亚型分类，提升了诊断精准度。

1. 基于图像数据的血液肿瘤分析诊断：血液肿瘤的图像数据主要涵盖外周血涂片、骨髓涂片和病理图像等。伴随AI的不断发展，基于深度学习的图像分析技术在血液肿瘤诊断中取得了显著进展，并正从单一模态图像数据诊断朝着多模态数据融合诊断的方向发展。Eckardt等[32]利用1 251例急性髓系白血病（AML）患者和236例健康骨髓捐献者的骨髓涂片图像数据，训练了一种深度学习模型。该模型能够自动且精确地分割骨髓图像中的细胞，较好地区分AML样本与健康对照组，预测NPM1基因的突变状态。模型平均受试者工作特征曲线下面积（AUROC）达到0.9699，说明模型分类性能较好。Miyoshi等[40]利用深度神经网络对388个全切片图像进行分析，构建了能够准确区分不同类型淋巴瘤的模型。该模型可对淋巴瘤的组织病理学图像进行分类，分类准确率高达97％。

尽管单模态图像分析技术在准确性和效率方面取得了进展，然而，单一模态无法捕捉患者之间复杂异质性的问题，影响了模型的准确性和泛化性。近年来，研究者开始尝试通过多模态图像整合分析的方式进一步提升诊断效果。李兰兰等[33]基于9 828张骨盆区域的正电子发射断层扫描（PET）和计算机断层扫描（CT）图像数据开发了一种融合多模态图像的卷积神经网络模型CNN-ViT。该模型通过整合PET和CT图像数据诊断弥漫大B细胞淋巴瘤（DLBCL）患者的骨髓受累情况。结果显示，该模型诊断DLBCL中骨髓受累的准确度、特异度、敏感度和F1分数分别是0.988、0.971、0.997和0.987，可以准确评估DLBCL骨髓受累情况。Xia等[41]整合了289例患者对比增强T1加权成像（CE-T1WI）、液体衰减反转恢复序列（FLAIR）序列图像以及从弥散加权成像（DWI）序列导出的表观扩散系数（ADC）图，开发了一种基于决策级融合的多参数卷积神经网络模型（DF-CNN），用于鉴别诊断原发性中枢神经系统淋巴瘤（PCNSL）与胶质母细胞瘤。在无须事先勾画肿瘤轮廓的情况下，该模型准确率达到0.899，高于基于CE-T1WI、FLAIR和ADC的单参数CNN模型（单参数模型准确率分别为0.884、0.782和0.700）。

这些基于图像数据的研究可提升血液肿瘤的精准诊断水平，同时在应用于血液肿瘤诊断的重要手段——血细胞形态学检查方面，AI应用进展迅速，已有各类AI辅助血细胞形态学识别的医疗器械应用于临床，并且正在走向规范化和标准化。在中国，为提升血液肿瘤诊断的准确性，已有研究者将AI应用于血液分析结果的审核和血细胞形态学的诊断[42]–[43]；并且有关专家团队已达成了血细胞形态学检查的中国专家共识[44]–[45]。

2. 基于分子数据的血液肿瘤分析诊断：血液肿瘤的分子数据主要包括基因突变、基因表达、DNA甲基化和蛋白质组学等数据类型。AI通过对上述数据的整合分析，深入解析血液肿瘤异质性特征，精准定义不同分子亚型。Li等[46]提出了一种结合自适应稀疏组套索惩罚的逻辑回归方法（LR-ASGL），解决了肿瘤基因表达谱数据中的噪声、基因分组和适应性基因选择等问题。通过实验验证，对急性白血病的平均预测准确率为97.2％，预测准确率高于弹性网络等模型，说明LR-ASGL对肿瘤分子诊断的有效性。Krali等[47]对1 131例北欧地区的急性淋巴细胞白血病（ALL）患者进行研究，结合DNA甲基化数据和基因表达数据开发机器学习模型ALLIUM，对ALL的17种分子亚型（包括HeH、ETV6∷RUNX1、T-ALL和PAX5alt等）进行分类。Xiong等[34]对128例自然杀伤T细胞淋巴瘤（NKTCL）样本进行了多组学研究，通过对基因表达进行无监督聚类等定义了TSIM、HEA、MB三种亚型，各亚型在细胞起源、转录特征、治疗反应方面有显著差异，为精准诊疗提供重要科学依据。上述研究应用AI技术优化血液肿瘤的分子亚型分类，显著提升了诊断精度，推动了分子诊断在血液肿瘤精准医疗中的应用。

此外，针对流式细胞术数据分析，除了使用传统机器学习方法外，深度学习技术也得到应用，这些技术的应用可实现血液肿瘤的自动化诊断与免疫特征分析[48]–[49]。AI在图像、分子以及流式细胞术数据的研究和应用推动了基于MICM的血液肿瘤精准诊断。

二、AI在血液肿瘤精准治疗方面的应用

AI在血液肿瘤治疗方面的应用主要集中在治疗响应预测和CDSS应用方面。在治疗响应预测方面，AI融合了多模态数据，提升了治疗响应预测水平；在CDSS方面，AI整合了临床和生物信息，优化数据分析流程，为个性化治疗决策提供重要支持。

1. AI在血液肿瘤治疗响应预测中的应用：血液肿瘤治疗响应预测可帮助医师选择有效的治疗方法，优化治疗效果，减少不良反应，实现个性化精准治疗。AI技术通过对血液肿瘤的临床数据、分子数据、影像数据等数据的整合分析，提升治疗响应预测的准确性。Jemaa等[35]依据Lugano 2014标准，建立基于深度学习的全自动化治疗反应评估算法，可自动对PET/CT数据进行代谢反应评估。对多个独立研究队列的分析显示，该方法与放射科医师的评定反应呈现高度的一致性，缩短医师在审查算法输出结果时所需的时间，具有整合到放射学工作流程中的潜力。Lee等[50]利用深度学习技术结合临床数据和病理学图像数据开发了一个多模态预测模型，通过对216例接受利妥昔单抗、环磷酰胺、多柔比星、长春新碱和泼尼松（R-CHOP）方案治疗的DLBCL患者的251张组织病理学切片图像进行分析，预测接受R-CHOP方案治疗的DLBCL患者对免疫化疗的反应，预测模型的AUROC值为0.856，说明了模型的有效性。Ali等[51]整合61例使用甲氨蝶呤的PCNSL患者的临床数据和磁共振图像（MRI）数据，使用梯度提升模型提取重要特征，采用支持向量机（SVM）对甲氨蝶呤治疗反应进行预测，平均测试准确率为（81.1±12.3）％，比仅使用临床特征的模型的准确率高20％，该模型可有效预测PCNSL患者使用甲氨蝶呤的疗效反应。Shen等[52]和Zhang等[53]通过对35个基因（TP53、MYD88、NOTCH1、NOTCH2、EZH2、SGK1等）的突变和3个基因（BCL2、BCL6、MYC）重排进行分析，构建了LymphPlex模型，将DLBCL分为七个遗传亚型。基于LymphPlex模型的结果，指导不同靶向药物和R-CHOP方案联合使用（R-CHOP-X），在GUIDANCE-01随机对照试验中进行了验证，R-CHOP-X方案的完全应答率、两年无进展生存（PFS）和总生存（OS）率均明显高于R-CHOP方案。LymphPlex模型为基于分子特征的靶向治疗提供重要科学依据，具有较好的临床可行性。这些研究表明，在血液肿瘤治疗中，AI应用于分子分型、多模态数据整合，可显著提升血液肿瘤治疗响应的预测精度，推动了个性化治疗的发展，增强了临床决策的效率。

2. AI决策支持系统在血液肿瘤治疗中的应用：决策支持系统是一类为决策者提供信息、模型和工具，帮助在复杂的情境中做出有效决策的信息系统[54]。在血液肿瘤治疗中，CDSS通过整合患者的临床数据、基因组信息、治疗方案、预后模型等，为临床医师提供个性化的决策建议[55]。目前，AI在血液肿瘤CDSS方面的应用主要表现为利用基于数据驱动的强化学习以及大规模语言模型技术。Herishanu等[56]基于最新医学指南、前沿研究、专家经验以及机器学习算法，开发了一个专用于慢性淋巴细胞白血病（CLL）治疗决策支持与疗效预测的临床决策支持系统。系统通过计算机向交互式智能聊天机器人提供与CLL治疗相关因素或从医疗文件中提取数据，生成一份详细的治疗需求、推理过程及替代方案的报告，为医师提供精准且全面的治疗建议。Shain等[36]基于强化学习算法利用800余例患者的临床数据（包括人口统计学信息、疾病分期、风险评估及完整的治疗过程数据）以及CD138阳性细胞的分子分析数据（如全外显子组测序和RNA测序等数据）开发了一种MM治疗策略的优化模型。该模型不仅能整合多维度的临床和分子数据，还具备动态调整治疗方案的能力，通过强化学习算法的自我学习和不断优化，能够根据患者个体的特征和治疗反应为临床决策提供支持。Lee等[37]基于GPT-4开发了一个知识驱动的大语言模型系统SEETrials，该系统主要由预处理、提示工程和后处理3个模块组成。其中，预处理模块能从美国临床肿瘤学会（ASCO）、美国血液学会（ASH）及PubMed数据库收集来自不同药物组的245个MM的临床试验摘要；提示工程模块能创建量身定制的提示，使数据能够全面且精确地提取和整合，最终指导大语言模型有效识别和合并相关试验的结果；后处理模块对提取的结果数据进行细化和结构化，以适应后续数据分析任务的要求。SEETrials能从精简和复杂医学文本中提取数据，在处理年度会议摘要方面能节省80％以上的时间，这使得医疗和研究人员能快速获得最新的临床见解，支持医师的治疗决策。同时，SEETrials还具备良好的泛化能力，可有效地从不同癌症类型中提取试验信息，并在各种疾病临床试验研究中获得较高的评价指标（F1分数为0.979～0.992）。

三、AI在血液肿瘤预后预测方面的应用

AI在血液肿瘤治疗的预后预测中的应用，主要集中在基于图像数据和分子数据的预后预测。在图像数据方面，AI融合了图像数据和临床数据，提升了预后预测的准确性；在分子数据方面，AI挖掘了高维分子数据中潜在的重要信息，鉴定出潜在的治疗靶点。

1. 基于图像数据的血液肿瘤预后预测：目前，血液肿瘤的预后图像数据主要有PET/CT图像和病理图像等，基于深度学习的图像特征提取技术以及多模态融合技术正被应用于血液肿瘤的预后预测。Jiang等[38]利用深度学习技术从PET图像中提取的深度学习评分（DLS），开发了一种新的预后图像生物标志物。这些标志物在预测PFS和OS方面显示出显著相关性，DLS训练集的PFS和OS的C-index分别为0.866和0.835，在两个外部验证队列中，PFS和OS的C-index分别为0.760和0.770、0.748和0.766，均超过了传统的国际预后指数（IPI）临床模型，表明该多参数模型的有效性。Driessen等[57]利用使用三种治疗方案的182例复发/难治性经典霍奇金淋巴瘤（R/R cHL）患者的数据（113例训练队列，69例验证队列），基于^18^F-氟代脱氧葡萄糖正电子发射断层扫描（^18^F-FDG PET）影像和临床特征，采用SUV4.0方法进行分割，从^18^F-FDG PET影像中提取代谢肿瘤体积（MTV）、所有病变距离之和（Spread）和肿瘤-肝脏SUV_mean_的比值（TLR_SUVmean_）放射组学特征，并结合B症状和复发/难治状态临床特征，使用反向选择和逻辑回归算法，开发了一个预测PFS的预后模型。模型在训练队列和验证队列中的AUC分别为0.81（95％ *CI* 0.805～0.814）和0.75（95％ *CI* 0.627～0.872），显著优于仅使用临床特征的模型（AUC分别为0.729和0.677，*P*<0.01）。罗与等[58]基于90例NKTCL患者的^18^F-FDG PET/CT影像数据，开发了一种融合临床和影像组学特征的复合预后预测模型。该模型结合PET和CT图像特征及临床危险因素，用于预测患者的无事件生存期。结果显示，复合模型在训练组和验证组的AUC分别为0.882、0.720，均高于单独的临床模型和影像组学模型。表明复合模型可以提供更全面的预后信息，为NKTCL患者提供了无创的风险分层工具，有助于个体化治疗。上述研究借助AI深度学习和多模态融合技术，在自动分割、特征提取以及预后分层等方面表现出色，能够较准确地预测PFS和OS等生存时间。

2. 基于分子数据的血液肿瘤预后预测：血液肿瘤预后的分子数据主要涵盖RNA测序、微阵列芯片等数据。AI技术能够从上述数据中提取出基因表达和疾病进展相关的关键特征进而构建预后风险模型。Zamani-Ahmadmahmudi等[59]通过SVM、对角线性判别分析（DLDA）、混合协变量分类模型（CCP）、贝叶斯混合协变量预测算法（BCC）、K近邻算法（KNN）和最邻质心算法（NC）六种机器学习方法筛选出与MM生存显著相关的关键基因（CCT2、CKS1B、PRKDC、NONO和UBE2A）。基于这些基因，在TCGA的MM RNA测序数据集（MMRF-CoMMpass，*n*＝412）中训练了一个预后预测模型，并在五个独立数据集（GSE2658、GSE13624、GSE9782、GSE6477和GSE57317，包含1 461例MM病例）中进行了外部验证。结果显示，该方法筛选出的基因靶点能够稳健地区分低风险和高风险患者（*P*≤0.001），并在预测能力上显著优于常规MM预后因素（如β_2_-微球蛋白、白蛋白、年龄和性别）（*P*≤0.01）。He等[39]收集来自GEO数据库的412例患者的mRNA数据和来自TCGA数据库的47例患者mRNA数据和临床数据，从877个与代谢相关的基因（MAG）通过单因素Cox回归分析筛选出92个预后相关候选MAG。基于上述MAG通过聚类分析将患者分为两种与代谢相关的亚型，这两个亚型在预后和免疫微环境上差异显著。最后，通过LASSO回归选择了14个MAG，并构建了预后风险模型，有效区分高低风险患者，并自动预测DLBCL患者的生存期。该模型在外部验证队列中1、2、3年OS率的AUC分别为0.78、0.61和0.61，显示出模型的稳健性和有效性。Tang等[60]收集来自GEO数据库的198个正常样本和2 322个AML样本的基因芯片数据，先后进行差异表达分析、主成分分析、富集分析等得到9个细胞表面标志物，构建AML患者风险分层的预后模型，该模型在GSE71014验证集中预测1、3、5年OS率的AUC分别为0.70、0.68和0.59，为预测AML患者的预后提供有价值的信息。上述研究通过利用AI对RNA-Seq和微阵列芯片等分子数据进行分析，鉴定新型分子标志和潜在治疗靶点，建立预后风险模型，成功识别出关键基因和高风险患者群体。

此外，利用AI技术，基于流式细胞术数据的分析，以及结合临床常规检测（如血液学和生化指标）与分子数据，构建的预后模型，为患者的风险分层和个性化治疗提供了支持[61]–[62]。同时，AI在图像、分子和多模态数据中的研究，进一步推动了血液肿瘤预后预测的发展。

四、总结

1. AI在血液肿瘤精准诊疗领域应用的优势：

（1）AI提升了血液肿瘤诊疗的准确性：从文献分析来看已有研究主要通过对图像数据和分子数据的处理，采用机器学习和深度学习，构建AI模型。研究普遍认为AI能够从医疗数据中提取有效信息，提升诊断和治疗的精准性[32]。随着多模态技术的发展，融合了多模态数据的AI模型，能够结合多学科知识，深入解析基因组与表型的关联，提高诊断的全面性和准确性[41],[50]。

（2）AI提高了血液肿瘤的诊疗效率：AI驱动的CDSS能够提高诊疗效率，优化医疗流程。AI能够快速地处理和分析大量的医疗数据，提取其中的重要信息，为医师提供实时诊疗建议[37],[56]，为患者提供及时的医疗服务。AI的自动化数据分析和重复性任务处理能力减轻了医师的工作负担[35]，缩短诊断时间，并能评估治疗方案的安全性与有效性，降低不良反应风险。

（3）AI提高了血液肿瘤的个性化诊疗能力：测序技术的发展产生了大量的分子数据，AI通过深入挖掘不同肿瘤亚型细胞起源、转录特征和治疗反应方面的差异，能够鉴定新型分子标志和潜在治疗靶点，为精准诊疗提供了有力支持[34],[39]。同时，AI通过对基因测序数据进行处理和分析，根据患者的基因组数据和其他生物学特征，结合临床数据挖掘靶点基因，制定个性化的诊断和治疗方案，实现个性化医疗和精准治疗[52]–[53]。

2. AI在血液肿瘤精准诊疗应用面临的问题：目前AI在血液肿瘤应用的主要问题是临床数据质量不高、模型算法可解释性差以及临床实践应用不足。

AI模型预测的准确性和科学性与数据的数量和质量关系密切。目前公开数据集少，不同机构缺少统一数据采集标准，研究采用的数据集多来自单中心，存在样本量小[33]、代表性不够、不完整等问题，其中图像和临床数据更为稀缺。许多血液肿瘤是罕见疾病，其数据更加不足[58]，致使模型难以充分学习疾病特征，导致一定程度的选择偏倚，影响了AI模型应用的准确性和泛化能力。

随着血液肿瘤领域中多组学分子、医学影像和临床等高维数据的不断增加，深度学习算法已成为处理这些数据的核心工具，由于推理过程的不透明性，尤其是深度学习复杂的学习机制通常呈现出“黑箱”性质[63]，导致现有模型与疾病形成机理的结合不足，临床医师难以理解和解释其推理过程，从而对其结果保持谨慎态度，难以完全信赖。模型的可解释性差成为制约AI技术应用的瓶颈[64]。

尽管AI在血液肿瘤的精准诊疗和预后预测中已有不少研究成果，但大多数模型的实践应用还相对缺乏，特别是大规模、多中心临床试验的验证还较少。虽然这些模型在实验环境中表现良好，但在实际临床诊疗中却难以取得同样出色的效果[50]。此外，AI在血液肿瘤领域缺乏系统化的全流程研究和应用框架，研究和应用的分散性阻碍了模型向临床应用的有效转化。

3. AI在血液肿瘤精准诊疗应用中的发展趋势：随着研究的不断深入，临床数据采集标准的建立、融合多模态信息的精准诊疗、模型算法系统化的临床验证成为该领域未来的发展趋势。

针对不同血液肿瘤建立统一且共享的数据标准和数据库，有助于整合多机构、多模态数据，大幅拓展数据的覆盖范围，增加其多样性，尤其为罕见血液肿瘤提供强有力的支撑。全面统一的数据质量控制体系还可提高数据准确性，有效降低误诊和漏诊风险。

多模态融合技术是AI的重要组成，可有效解决单一模态无法捕捉复杂异质性的问题。随着测序技术的不断发展，空间转录组学、单细胞组学、表观基因组学等新的医疗数据不断涌现，融合多模态信息的AI技术，可更深入地理解复杂的基因组-表型关联，为血液肿瘤的精准诊疗和预后预测提供有力支持。

模型算法系统化的临床验证可有效提高AI 模型的应用效果。通过多中心临床试验对模型的安全性、稳定性和泛化能力进行评估，以确保其在临床中的有效性。通过构建从AI模型研发到临床应用的全流程体系，借助持续的临床反馈与优化机制，实现模型的不断迭代更新，提升其可靠性，确保模型适用血液肿瘤中不同的患者群体和临床环境。
